# *N*′-[(*Z*)-4-Methyl­benzyl­idene]-4-nitro­benzohydrazide monohydrate

**DOI:** 10.1107/S1600536808037008

**Published:** 2008-11-13

**Authors:** Hoong-Kun Fun, Samuel Robinson Jebas, K. V. Sujith, B. Kalluraya

**Affiliations:** a X-ray Crystallography Unit, School of Physics, Universiti Sains Malaysia, 11800 USM, Penang, Malaysia; b Department of Studies in Chemistry, Mangalore University, Mangalagangotri, Mangalore 574 199, India

## Abstract

In the title compound, C_15_H_13_N_3_O_3_·H_2_O, the two benzene rings form a dihedral angle of 2.03 (2)°. In the crystal structure, adjacent hydrazide mol­ecules are linked into dimers by water mol­ecules; these dimers are then stacked along the *b* axis. Inter­molecular O—H⋯O, O—H⋯N and C—H⋯O hydrogen bonds and a π–π stacking inter­action between the nitro­benzene and tolyl rings with a centroid–centroid distance of 3.8208 (3) Å are observed. There is also a short O⋯N contact [2.6824 (7) Å].

## Related literature

For related literature on hydrazones, see: Sridhar & Perumal (2003 ▶). For the biological applications of hydrazides/hydrazones, see: Bedia *et al.* (2006 ▶). For a related structure, see: Fun *et al.* (2008 ▶). For bond-length data, see: Allen *et al.* (1987 ▶).
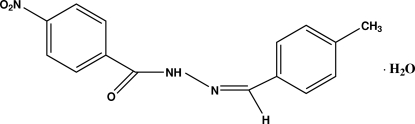


## Experimental

### 

#### Crystal data

C_15_H_13_N_3_O_3_·H_2_O
*M* *_r_* = 301.30
Triclinic, 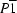

*a* = 6.5387 (1) Å
*b* = 6.9730 (1) Å
*c* = 15.9064 (3) Å
α = 80.524 (1)°
β = 82.628 (1)°
γ = 85.036 (1)°
*V* = 707.85 (2) Å^3^
*Z* = 2
Mo *K*α radiation
μ = 0.10 mm^−1^
*T* = 100.0 (1) K
0.68 × 0.44 × 0.23 mm

#### Data collection

Bruker SMART APEXII CCD area-detector diffractometer
Absorption correction: multi-scan (**SADABS**; Bruker, 2005 ▶) *T*_min_ = 0.932, *T*_max_ = 0.976
31311 measured reflections
7380 independent reflections
6571 reflections with *I* > 2σ(*I*)
*R*_int_ = 0.020

#### Refinement

*R*[*F* ^2^ > 2σ(*F* ^2^)] = 0.037
*wR*(*F* ^2^) = 0.118
*S* = 1.05
7380 reflections
211 parameters
4 restraints
H atoms treated by a mixture of independent and constrained refinement
Δρ_max_ = 0.48 e Å^−3^
Δρ_min_ = −0.59 e Å^−3^

### 

Data collection: *APEX2* (Bruker, 2005 ▶); cell refinement: *SAINT* (Bruker, 2005 ▶); data reduction: *SAINT*; program(s) used to solve structure: *SHELXTL* (Sheldrick, 2008 ▶); program(s) used to refine structure: *SHELXTL*; molecular graphics: *SHELXTL*; software used to prepare material for publication: *SHELXTL* and *PLATON* (Spek, 2003 ▶).

## Supplementary Material

Crystal structure: contains datablocks global, I. DOI: 10.1107/S1600536808037008/is2358sup1.cif

Structure factors: contains datablocks I. DOI: 10.1107/S1600536808037008/is2358Isup2.hkl

Additional supplementary materials:  crystallographic information; 3D view; checkCIF report

## Figures and Tables

**Table 1:** Hydrogen-bond geometry (Å, °)

*D*—H⋯*A*	*D*—H	H⋯*A*	*D*⋯*A*	*D*—H⋯*A*
N2—H1*N*2⋯O1*W*	0.864 (8)	1.978 (9)	2.8191 (7)	164.4 (11)
O1*W*—H2*W*1⋯O1^i^	0.837 (9)	2.013 (9)	2.8327 (7)	166.1 (11)
O1*W*—H1*W*1⋯O1^ii^	0.851 (9)	2.258 (11)	2.9430 (6)	137.7 (10)
O1*W*—H1*W*1⋯N1^ii^	0.851 (9)	2.357 (9)	3.1287 (7)	151.0 (11)
C1—H1*A*⋯O1*W*^iii^	0.93	2.50	3.4090 (7)	165
C4—H4*A*⋯O2^iv^	0.93	2.58	3.4565 (8)	157
C7—H7*A*⋯O1*W*	0.93	2.55	3.2393 (7)	132

Symmetry codes: (i) 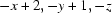; (ii) 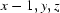; (iii) 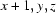; (iv) 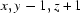.

**Fig. 1.**
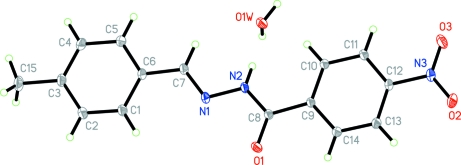
The molecular structure of the title compound, showing 50% probability displacement ellipsoids and the atom numbering scheme.

**Fig. 2.**
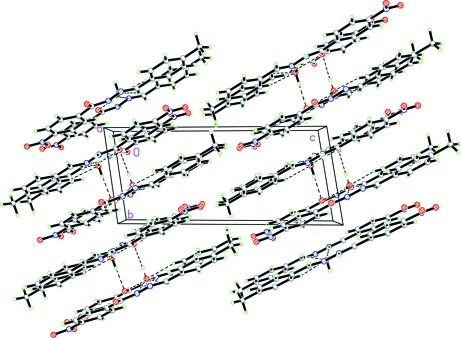
The crystal packing of the title compound, viewed down the a axis, showing stacking of the dimers along the b axis.

## supplementary crystallographic information

### Comment

Hydrazones are versatile intermediates and important building blocks. Aryl hydrazones are important building blocks for the synthesis of a variety of heterocyclic compounds such as pyrazolines and pyrazoles (Sridhar & Perumal, 2003). Hydrazones of aliphatic and aromatic methyl ketones yield pyrazole-4-carboxaldehyde on diformylation by the treatment with Vilsmeier reagent. A series of hydrazide-hydrazones were reported to possess good antituberculosis activity (Bedia *et al.*, 2006). Prompted by these review and in continuation of our work (Fun *et al.*, 2008), we here in report the crystal structure of the title compound, (I).

Bond lengths and angles in (I) (Fig. 1) are found to have normal values (Allen *et al.*, 1987). The two benzene rings are essentially planar with the maximum deviation from planarity being -0.004 (1) Å for atom C6 and 0.002 (1) Å for atom C12, respectively. The dihedral angle formed by the benzene (C1—C6) and (C9—C14) rings is 2.03 (2)°.

The crystal packing is consolidated by O—H···O, O—H···N, C—H···O and N—H···O inter and intramolecular hydrogen bonding (Table 1). Furthermore, the packing is strengthened by π-π stacking interactions involving the benzene (C1—C6) (*Cg*1) and the symmetry related (C9—C14) ring (*Cg*2) [*Cg*1···*Cg*2^i^ = 3.8208 (3) Å; symmetry code: (i) 2-*x*, 1-*y*, -*z*] together with O···N short contacts [2.6824 (7) Å]. In the crystal packing, adjacent molecules are linked into dimers by water molecules and the dimers were then stacked down the [010] direction (Fig. 2).

### Experimental

The title compound, C_15_H_15_N_3_O_4_, was obtained by refluxing 4-nitrobenzhydrazide (0.01 mol) and 4-methylbenzaldehyde (0.01 mol) in ethanol (30 ml) by adding 3 drops of concentrated sulfuric acid for 3 h. Excess ethanol was removed from the reaction mixture under reduced pressure. The solid product obtained was filtered, washed with water and dried. Crystals suitable for X-ray analysis were obtained from ethanol by slow evaporation.

### Refinement

The amino and water H atoms were located in a difference map and refined with restraints of N—H = 0.85 (1) Å and O—H = 0.84 (1) Å. The remaining H atoms were positioned geometrically [C—H = 0.93 Å (aromatic) or 0.96 Å (methyl)] and refined using a riding model, with *U*_iso_(H) = 1.2*U*_eq_(aromatic C) and 1.5*U*_eq_(methyl C). A rotating group model was used for the methyl group.

### Crystal data

**Table:**

C_15_H_13_N_3_O_3_·H_2_O	*Z* = 2
*M**_r_* = 301.30	*F*_000_ = 316
Triclinic, *P*1	*D*_x_ = 1.414 Mg m^−^^3^
Hall symbol: -P 1	Mo *K*α radiation λ = 0.71073 Å
*a* = 6.5387 (1) Å	Cell parameters from 9969 reflections
*b* = 6.9730 (1) Å	θ = 2.6–26.3º
*c* = 15.9064 (3) Å	µ = 0.11 mm^−^^1^
α = 80.524 (1)º	*T* = 100.0 (1) K
β = 82.628 (1)º	Block, colourless
γ = 85.036 (1)º	0.68 × 0.44 × 0.23 mm
*V* = 707.85 (2) Å^3^	

### Data collection

**Table:**

Bruker SMART APEXII CCD area-detector diffractometer	7380 independent reflections
Radiation source: fine-focus sealed tube	6571 reflections with *I* > 2σ(*I*)
Monochromator: graphite	*R*_int_ = 0.020
*T* = 100.0(1) K	θ_max_ = 37.5º
φ and ω scans	θ_min_ = 2.6º
Absorption correction: multi-scan(*SADABS*; Bruker, 2005)	*h* = −11→11
*T*_min_ = 0.932, *T*_max_ = 0.976	*k* = −11→10
31311 measured reflections	*l* = −27→27

### Refinement

**Table:**

Refinement on *F*^2^	Secondary atom site location: difference Fourier map
Least-squares matrix: full	Hydrogen site location: inferred from neighbouring sites
*R*[*F*^2^ > 2σ(*F*^2^)] = 0.037	H atoms treated by a mixture of independent and constrained refinement
*wR*(*F*^2^) = 0.119	*w* = 1/[σ^2^(*F*_o_^2^) + (0.0681*P*)^2^ + 0.1283*P*] where *P* = (*F*_o_^2^ + 2*F*_c_^2^)/3
*S* = 1.05	(Δ/σ)_max_ = 0.001
7380 reflections	Δρ_max_ = 0.48 e Å^−^^3^
211 parameters	Δρ_min_ = −0.59 e Å^−^^3^
4 restraints	Extinction correction: none
Primary atom site location: structure-invariant direct methods	

### Special details

**Table:**

Experimental. The data was collected with the Oxford Cyrosystem Cobra low-temperature attachment.
Geometry. All e.s.d.'s (except the e.s.d. in the dihedral angle between two l.s. planes) are estimated using the full covariance matrix. The cell e.s.d.'s are taken into account individually in the estimation of e.s.d.'s in distances, angles and torsion angles; correlations between e.s.d.'s in cell parameters are only used when they are defined by crystal symmetry. An approximate (isotropic) treatment of cell e.s.d.'s is used for estimating e.s.d.'s involving l.s. planes.
Refinement. Refinement of *F*^2^ against ALL reflections. The weighted *R*-factor *wR* and goodness of fit *S* are based on *F*^2^, conventional *R*-factors *R* are based on *F*, with *F* set to zero for negative *F*^2^. The threshold expression of *F*^2^ > σ(*F*^2^) is used only for calculating *R*-factors(gt) *etc*. and is not relevant to the choice of reflections for refinement. *R*-factors based on *F*^2^ are statistically about twice as large as those based on *F*, and *R*- factors based on ALL data will be even larger.

### Fractional atomic coordinates and isotropic or equivalent isotropic displacement parameters (Å^2^)

**Table:**

	*x*	*y*	*z*	*U*_iso_*/*U*_eq_	
O1	1.27580 (6)	0.77076 (7)	−0.04714 (3)	0.01642 (8)	
O2	0.75581 (8)	1.18850 (8)	−0.38841 (3)	0.02159 (10)	
O3	0.46360 (8)	1.16591 (9)	−0.30724 (3)	0.02477 (11)	
N1	1.08155 (7)	0.62939 (7)	0.10666 (3)	0.01228 (8)	
N2	0.97488 (7)	0.71707 (7)	0.03900 (3)	0.01180 (8)	
N3	0.65301 (8)	1.14188 (7)	−0.31919 (3)	0.01517 (9)	
C1	1.25937 (9)	0.43357 (8)	0.25978 (3)	0.01394 (9)	
H1A	1.3528	0.4556	0.2105	0.017*	
C2	1.32689 (9)	0.33958 (9)	0.33630 (4)	0.01558 (9)	
H2A	1.4660	0.2992	0.3375	0.019*	
C3	1.19044 (10)	0.30421 (9)	0.41173 (4)	0.01618 (10)	
C4	0.98236 (10)	0.36711 (9)	0.40850 (4)	0.01732 (10)	
H4A	0.8891	0.3455	0.4579	0.021*	
C5	0.91322 (9)	0.46166 (9)	0.33225 (4)	0.01511 (9)	
H5A	0.7744	0.5032	0.3313	0.018*	
C6	1.05015 (8)	0.49509 (8)	0.25695 (3)	0.01205 (8)	
C7	0.96681 (8)	0.59124 (8)	0.17866 (3)	0.01265 (9)	
H7A	0.8259	0.6261	0.1809	0.015*	
C8	1.08418 (8)	0.78209 (7)	−0.03665 (3)	0.01124 (8)	
C9	0.96283 (8)	0.87393 (7)	−0.10826 (3)	0.01078 (8)	
C10	0.74981 (8)	0.92155 (8)	−0.09685 (3)	0.01259 (9)	
H10A	0.6760	0.8932	−0.0428	0.015*	
C11	0.64812 (8)	1.01101 (8)	−0.16601 (3)	0.01320 (9)	
H11A	0.5067	1.0435	−0.1589	0.016*	
C12	0.76204 (8)	1.05088 (8)	−0.24589 (3)	0.01220 (8)	
C13	0.97361 (9)	1.00639 (8)	−0.25947 (3)	0.01340 (9)	
H13A	1.0465	1.0355	−0.3137	0.016*	
C14	1.07314 (8)	0.91711 (8)	−0.18969 (3)	0.01269 (9)	
H14A	1.2148	0.8857	−0.1972	0.015*	
C15	1.26554 (12)	0.20102 (11)	0.49388 (4)	0.02473 (13)	
H15A	1.1517	0.1899	0.5386	0.037*	
H15B	1.3687	0.2740	0.5096	0.037*	
H15C	1.3242	0.0733	0.4859	0.037*	
O1W	0.56488 (7)	0.60570 (7)	0.07749 (3)	0.01669 (8)	
H1N2	0.8430 (12)	0.7058 (17)	0.0471 (8)	0.027 (3)*	
H2W1	0.5907 (17)	0.4907 (12)	0.0684 (8)	0.031 (3)*	
H1W1	0.4410 (13)	0.6364 (17)	0.0668 (8)	0.035 (3)*	

### Atomic displacement parameters (Å^2^)

**Table:**

	*U*^11^	*U*^22^	*U*^33^	*U*^12^	*U*^13^	*U*^23^
O1	0.00972 (15)	0.0221 (2)	0.01583 (17)	0.00035 (13)	−0.00227 (13)	0.00155 (14)
O2	0.0250 (2)	0.0269 (2)	0.01076 (17)	−0.00148 (17)	−0.00275 (15)	0.00358 (15)
O3	0.01615 (19)	0.0361 (3)	0.0199 (2)	0.00133 (18)	−0.00737 (16)	0.00461 (19)
N1	0.01330 (18)	0.01250 (17)	0.01075 (17)	−0.00018 (13)	−0.00421 (13)	0.00081 (13)
N2	0.01085 (17)	0.01426 (18)	0.00977 (16)	−0.00046 (13)	−0.00339 (13)	0.00113 (13)
N3	0.0174 (2)	0.01585 (19)	0.01215 (18)	−0.00094 (15)	−0.00535 (15)	0.00083 (14)
C1	0.0137 (2)	0.0152 (2)	0.01227 (19)	−0.00081 (16)	−0.00285 (15)	0.00066 (15)
C2	0.0155 (2)	0.0166 (2)	0.0143 (2)	0.00085 (17)	−0.00481 (16)	−0.00018 (16)
C3	0.0208 (2)	0.0158 (2)	0.01158 (19)	0.00194 (17)	−0.00496 (17)	−0.00040 (16)
C4	0.0199 (2)	0.0196 (2)	0.01066 (19)	0.00142 (18)	−0.00093 (17)	0.00081 (16)
C5	0.0148 (2)	0.0174 (2)	0.01210 (19)	0.00041 (16)	−0.00163 (16)	−0.00002 (16)
C6	0.01369 (19)	0.01192 (19)	0.01037 (18)	−0.00115 (15)	−0.00323 (14)	0.00030 (14)
C7	0.01325 (19)	0.0133 (2)	0.01122 (18)	−0.00088 (15)	−0.00333 (15)	0.00004 (15)
C8	0.01101 (18)	0.01159 (18)	0.01084 (18)	0.00006 (14)	−0.00243 (14)	−0.00044 (14)
C9	0.01090 (18)	0.01125 (18)	0.00998 (17)	−0.00020 (14)	−0.00238 (14)	−0.00045 (14)
C10	0.01106 (18)	0.0154 (2)	0.01048 (18)	0.00026 (15)	−0.00182 (14)	0.00036 (15)
C11	0.01170 (19)	0.0154 (2)	0.01187 (19)	0.00029 (15)	−0.00297 (15)	0.00029 (15)
C12	0.01382 (19)	0.01256 (19)	0.01019 (18)	−0.00090 (15)	−0.00400 (15)	0.00040 (14)
C13	0.0141 (2)	0.0151 (2)	0.01041 (18)	−0.00143 (16)	−0.00148 (15)	−0.00005 (15)
C14	0.01159 (19)	0.0146 (2)	0.01130 (18)	−0.00053 (15)	−0.00128 (14)	−0.00060 (15)
C15	0.0321 (3)	0.0271 (3)	0.0134 (2)	0.0068 (2)	−0.0081 (2)	0.0011 (2)
O1W	0.01130 (16)	0.0212 (2)	0.01725 (18)	−0.00029 (13)	−0.00349 (13)	−0.00109 (14)

### Geometric parameters (Å, °)

**Table:**

O1—C8	1.2400 (6)	C6—C7	1.4605 (7)
O2—N3	1.2246 (7)	C7—H7A	0.9300
O3—N3	1.2298 (7)	C8—C9	1.4970 (7)
N1—C7	1.2881 (7)	C9—C14	1.3994 (7)
N1—N2	1.3859 (6)	C9—C10	1.3994 (7)
N2—C8	1.3491 (7)	C10—C11	1.3898 (7)
N2—H1N2	0.864 (8)	C10—H10A	0.9300
N3—C12	1.4703 (7)	C11—C12	1.3867 (7)
C1—C2	1.3896 (8)	C11—H11A	0.9300
C1—C6	1.4019 (8)	C12—C13	1.3884 (8)
C1—H1A	0.9300	C13—C14	1.3895 (7)
C2—C3	1.4008 (8)	C13—H13A	0.9300
C2—H2A	0.9300	C14—H14A	0.9300
C3—C4	1.3977 (9)	C15—H15A	0.9600
C3—C15	1.5035 (8)	C15—H15B	0.9600
C4—C5	1.3913 (8)	C15—H15C	0.9600
C4—H4A	0.9300	O1W—H2W1	0.837 (8)
C5—C6	1.3999 (8)	O1W—H1W1	0.851 (8)
C5—H5A	0.9300		
			
C7—N1—N2	114.21 (4)	O1—C8—N2	122.52 (5)
C8—N2—N1	118.48 (4)	O1—C8—C9	120.74 (5)
C8—N2—H1N2	125.4 (8)	N2—C8—C9	116.74 (4)
N1—N2—H1N2	115.2 (8)	C14—C9—C10	119.65 (5)
O2—N3—O3	123.96 (5)	C14—C9—C8	116.93 (4)
O2—N3—C12	118.18 (5)	C10—C9—C8	123.40 (5)
O3—N3—C12	117.85 (5)	C11—C10—C9	120.23 (5)
C2—C1—C6	119.91 (5)	C11—C10—H10A	119.9
C2—C1—H1A	120.0	C9—C10—H10A	119.9
C6—C1—H1A	120.0	C12—C11—C10	118.60 (5)
C1—C2—C3	121.56 (5)	C12—C11—H11A	120.7
C1—C2—H2A	119.2	C10—C11—H11A	120.7
C3—C2—H2A	119.2	C11—C12—C13	122.73 (5)
C4—C3—C2	118.18 (5)	C11—C12—N3	118.38 (5)
C4—C3—C15	120.88 (6)	C13—C12—N3	118.89 (5)
C2—C3—C15	120.95 (6)	C12—C13—C14	118.01 (5)
C5—C4—C3	120.72 (5)	C12—C13—H13A	121.0
C5—C4—H4A	119.6	C14—C13—H13A	121.0
C3—C4—H4A	119.6	C13—C14—C9	120.79 (5)
C4—C5—C6	120.80 (5)	C13—C14—H14A	119.6
C4—C5—H5A	119.6	C9—C14—H14A	119.6
C6—C5—H5A	119.6	C3—C15—H15A	109.5
C5—C6—C1	118.83 (5)	C3—C15—H15B	109.5
C5—C6—C7	118.05 (5)	H15A—C15—H15B	109.5
C1—C6—C7	123.11 (5)	C3—C15—H15C	109.5
N1—C7—C6	122.33 (5)	H15A—C15—H15C	109.5
N1—C7—H7A	118.8	H15B—C15—H15C	109.5
C6—C7—H7A	118.8	H2W1—O1W—H1W1	106.0 (10)
			
C7—N1—N2—C8	172.77 (5)	N2—C8—C9—C14	−171.45 (5)
C6—C1—C2—C3	−0.09 (9)	O1—C8—C9—C10	−168.59 (5)
C1—C2—C3—C4	−0.32 (9)	N2—C8—C9—C10	10.18 (8)
C1—C2—C3—C15	179.55 (6)	C14—C9—C10—C11	0.09 (8)
C2—C3—C4—C5	0.20 (9)	C8—C9—C10—C11	178.42 (5)
C15—C3—C4—C5	−179.67 (6)	C9—C10—C11—C12	0.20 (8)
C3—C4—C5—C6	0.33 (9)	C10—C11—C12—C13	−0.47 (8)
C4—C5—C6—C1	−0.73 (9)	C10—C11—C12—N3	178.76 (5)
C4—C5—C6—C7	178.66 (5)	O2—N3—C12—C11	175.31 (5)
C2—C1—C6—C5	0.61 (8)	O3—N3—C12—C11	−4.94 (8)
C2—C1—C6—C7	−178.75 (5)	O2—N3—C12—C13	−5.43 (8)
N2—N1—C7—C6	178.63 (5)	O3—N3—C12—C13	174.32 (6)
C5—C6—C7—N1	179.37 (5)	C11—C12—C13—C14	0.42 (8)
C1—C6—C7—N1	−1.26 (9)	N3—C12—C13—C14	−178.81 (5)
N1—N2—C8—O1	−1.88 (8)	C12—C13—C14—C9	−0.10 (8)
N1—N2—C8—C9	179.38 (4)	C10—C9—C14—C13	−0.14 (8)
O1—C8—C9—C14	9.78 (8)	C8—C9—C14—C13	−178.58 (5)

### Hydrogen-bond geometry (Å, °)

**Table:**

*D*—H···*A*	*D*—H	H···*A*	*D*···*A*	*D*—H···*A*
N2—H1N2···O1W	0.864 (8)	1.978 (9)	2.8191 (7)	164.4 (11)
O1W—H2W1···O1^i^	0.837 (9)	2.013 (9)	2.8327 (7)	166.1 (11)
O1W—H1W1···O1^ii^	0.851 (9)	2.258 (11)	2.9430 (6)	137.7 (10)
O1W—H1W1···N1^ii^	0.851 (9)	2.357 (9)	3.1287 (7)	151.0 (11)
C1—H1A···O1W^iii^	0.93	2.50	3.4090 (7)	165
C4—H4A···O2^iv^	0.93	2.58	3.4565 (8)	157
C7—H7A···O1W	0.93	2.55	3.2393 (7)	132

Symmetry codes: (i) −*x*+2, −*y*+1, −*z*; (ii) *x*−1, *y*, *z*; (iii) *x*+1, *y*, *z*; (iv) *x*, *y*−1, *z*+1.
